# Beauty, Bias, and Beyond: The Role of Artificial Intelligence in Aesthetic Medicine

**DOI:** 10.1111/jocd.70853

**Published:** 2026-04-07

**Authors:** Samuel A. Knoedler, Michael Alfertshofer, Bruna S. F. Bravo, Giuseppe Sofo, Francesco Mazzarone, Robert Gaudin, Hermann F. Sailer, Alexandre G. Lellouch, Leonard Knoedler

**Affiliations:** ^1^ Department of Plastic Surgery and Hand Surgery, Klinikum Rechts der Isar Technical University of Munich Munich Germany; ^2^ Instituto Ivo Pitanguy, Hospital Santa Casa de Misericórdia Rio de Janeiro Pontifícia Universidade Católica do Rio de Janeiro Brazil; ^3^ Charité – Universitätsmedizin Berlin, Corporate Member of Freie Universität Berlin, Humboldt‐Universität Zu Berlin, and Berlin Institute of Health Department of Oral and Maxillofacial Surgery Berlin Germany; ^4^ Department of Dermatology Bravo Private Clinic Rio de Janeiro Brazil; ^5^ Department of Oral and Maxillofacial Surgery SailerClinic Zurich Switzerland; ^6^ Division of Plastic and Reconstructive Surgery Cedars‐Sinai Medical Center Los Angeles California USA; ^7^ AP‐HP, Hôpital Européen Georges Pompidou Hematology Department Paris France; ^8^ Université Paris Cité Inserm the Paris Cardiovascular Research Center, Team Endotheliopathy and Hemostasis Disorders Paris France

**Keywords:** aesthetic assessment, aesthetic medicine, AI, artificial intelligence, treatment refinement

To the Editor,

We read with great interest the recently published article “Artificial Intelligence in Aesthetic Medicine: Applications, Challenges, and Future Directions” by Al‐Dhubaibi et al. [1]. Its comprehensive overview of AI‐assisted analysis, robotic procedures, and predictive modeling underscores both the promise and the complexity of integrating these technologies into an inherently artistic field [2]. Motivated by these insights, we aim to offer additional perspective on the opportunities and limitations of AI as it enters everyday aesthetic practice, with particular emphasis on the balance between algorithmic analysis and the subjective, human‐centered nature of aesthetic decision‐making.

Aesthetic medicine is undergoing a profound technological shift. AI may evoke images of futuristic robots and soulless machines, seemingly far removed from the human‐centered craft of aesthetic care. Yet, it is already here, embedded in facial analysis tools, outcome prediction models, and postoperative monitoring systems. This accelerating interspersion raises essential questions: can AI truly grasp something as nuanced and subjective as human beauty? [3] What do we gain or risk losing when algorithms enter a domain shaped by intuition and artistry? And, perhaps most importantly, is this shift genuinely for the better?

The strengths of AI lie first in its analytical accuracy. Machine‐learning algorithms can detect subtle asymmetries, evaluate skin characteristics on a pixel level, and simulate postoperative outcomes with a degree of reproducibility unattainable by human appraisal alone. Such tools improve diagnostic rigor and help patients form realistic expectations by visualizing probable results before a procedure is performed. Similarly, dermatologic applications of AI allow highly individualized treatment strategies driven by complex, multimodal datasets [4, 5]. These systems integrate variables such as skin type, genetic predisposition, and long‐term behavior patterns to generate tailored regimens, thereby reducing trial‐and‐error and enhancing safety. Furthermore, AI improves efficiency by automating labor‐intensive tasks, streamlining image analysis, and offering real‐time feedback during procedures. Predictive analytics can flag elevated risk for swelling, asymmetry, or scarring, prompting earlier clinical intervention and strengthening informed consent. When properly integrated, AI therefore augments clinical decision‐making, elevates standardization, and may meaningfully reduce complication rates.

Despite these benefits, the clinical deployment of AI raises critical concerns. Aesthetic medicine is not purely technical, it is deeply subjective [2, 3]. Beauty is culturally constructed, psychologically meaningful, and often defined by irregularities rather than symmetry alone. AI‐driven recommendations, if trained on narrow datasets, risk reinforcing a homogenized aesthetic ideal [6, 7]. This is particularly problematic in ethnically diverse populations where facial proportions, beauty norms, and cultural expectations vary widely. Without deliberate inclusion of diverse training data, algorithms may inadvertently marginalize non‐Western phenotypes or misinterpret skin pathology in darker skin types. Equally significant is the potential erosion of the clinician–patient relationship. Overreliance on AI could shift consultations from nuanced conversations to data‐generated directives, reducing the space for empathy and individualized discussion. Aesthetic decisions often hinge not on morphology but on emotion, identity, and personal narrative—dimensions no algorithm can fully capture. Ethical issues further complicate adoption. Biometric data carry substantial privacy risks, and opaque “black‐box” systems undermine transparency and trust [8]. Robust regulation and explainable AI are therefore indispensable. Improving dataset diversity, increasing algorithmic transparency, and maintaining mandatory human oversight in clinical decision‐making may help mitigate these risks and ensure that AI supports rather than replaces individualized patient care.

By handling data‐heavy tasks such as preoperative imaging, risk prediction, postoperative monitoring, AI frees practitioners to focus on the human and artistic dimensions of their work [9]. Current AI applications often serve more as patient‐engagement tools than mature diagnostic instruments, reflecting their novelty and limited generalizability. Human expertise remains irreplaceable: no algorithm can dictate the ideal filler volume or incision angle; such decisions rest on years of experiential knowledge. Regulatory frameworks appropriately mandate that a qualified human make the final clinical judgment. Another major challenge remains generalizability. Reliable aesthetic assessment requires vast, diverse datasets and must account for subtle, culturally influenced beauty cues that are difficult to quantify. AI still struggles with these subjective nuances and remains sensitive to data variation, bias, and limited interpretability. At the same time, AI enables complex surgical simulations and clearer patient education [10]. It helps surgeons understand individual expectations and deliver predictions based on real anatomy and realistic surgical possibilities. Eventually, practitioners may develop personalized AI systems trained on their own techniques and outcomes, ushering in individualized rather than universalized AI.

Quo vadis? As AI evolves, so must the professionals who use it. Integrating these tools ethically and effectively requires continuous education, interdisciplinary collaboration, and a commitment to patient‐centered care [11]. The rise of AI presents both opportunities and risks; the challenge is achieving a balance in which technology enhances, rather than erodes, the human essence of aesthetic medicine. After all, the beauty of our field lies not in algorithmic perfection, but in the uniquely human ability to understand, create, and care.

## Author Contributions

S.A.K., M.A., and L.K. conceived the idea and designed the concept of the commentary. S.A.K., M.A., and B.S.F.B. performed the literature review and drafted the initial manuscript. G.S., F.M., and R.G. contributed to the critical discussion of clinical implications and integration of aesthetic perspectives. H.F.S., F.M., R.G., A.G.L. provided expert insights on the ethical, translational, and AI‐related aspects. L.K. and R.G. critically revised the manuscript for intellectual content and coherence. All authors contributed to manuscript editing, reviewed, and approved the final version of the article.

## Funding

The authors have nothing to report.

## Conflicts of Interest

The authors declare no conflicts of interest.

## Data Availability

Data sharing not applicable to this article as no datasets were generated or analysed during the current study.
